# Hospital Outcomes of Adult Diabetic Patients by Glycated Hemoglobin Level in Nonsurgical Pathology in a High-Complexity Institution

**DOI:** 10.1177/1179551419882676

**Published:** 2019-10-16

**Authors:** German Camilo Giraldo-Gonzalez, Cristian Giraldo-Guzman, Abelardo Montenegro-Cantillo, Angie Carolina Andrade-García, Duvan Snaider Duran-Ardila, David Felipe Grisales-Salazar, Sara Camila Castiblanco-Arroyave

**Affiliations:** 1 Fundación Valle del Lili, Instituto de investigaciones clinicas, ICESI University, Cali, Colombia; 2 Universidad de Caldas, Internal Medicine Department, Manizales, Colombia; 3 Universidad de Manizales, Clinica Versalles, Internal Medicine Department, Manizales, Colombia; 4 Universidad de Manizales, Internal Medicine Department, Manizales, Colombia

**Keywords:** Diabetes mellitus type 2, glycated hemoglobin A, HbA1c, inpatient, diabetes complications, patient readmission

## Abstract

Recent evidence supports the relationship between in-hospital hyperglycemia and inpatient complications. Besides, glycated hemoglobin (HbA1c) can predict the clinical course of patients with type 2 diabetes mellitus (DM2) during hospital stays. This study aimed to assess the relationship between HbA1c levels and inpatient outcomes. Type 2 diabetes mellitus patients with age greater than 18 years, hospital length of stay greater than 24 hours, and one HbA1c report during their in-hospital management were included. All the electronic care records of patients admitted at the Clinical Versalles, a high-volume institution, in Manizales-Colombia were revised. The following variables were considered: hospital length of stay, diagnoses at the arrival, complications, capillary glucose levels, and treatment at discharge. Variables were categorized by HbA1c levels: group 1 = ⩽ 7%, group 2 = 7.01% to 8.5%, group 3 = 8.51% to ⩽10% and group 4 = >10%. There were a total of 232 patients. Average age was 69.7 years, mean HbA1c was 7.19 ± 2.03, average body mass index (BMI) was 28.8 ± 5.6. About HbA1c, 146 (62.9%) had ⩽7.5%. The most frequent admission diagnosis was by cardiovascular diseases. Average hospitalization was 7.5 ± 5.7 days. There was no relationship between the levels of HbA1c with hospital stays, inpatient complications, or readmissions. Infections and respiratory diseases were more common conditions related to higher HbA1c levels, especially when these were 8.5%. In diabetic patients with nonsurgical diseases and high HbA1c levels, there was no association with clinical complications, length of stay, readmissions, or in-hospital mortality, but changes in treatment at discharge were observed.

## Introduction

Chronic hyperglycemia is the main expression of type 2 diabetes mellitus (DM2), which is among the most costly diseases in both Latin America and around the world.^1^ In Colombia, the prevalence of DM2 in the adult population is 7% to 9%.^2^ Outpatient inadequate management of this disease results in increased morbidity and mortality with higher hospitalization rates and longer hospital stays.^3-5^ For chronic prevention of adverse outcomes, glycated hemoglobin (HbA1c) is used and targeted, looking for less diabetes morbidity complications. Some studies have shown that strict outpatient control (goal HbA1c of 6.0%) is associated with an increase in mortality.^6,7^ On the contrary, it has been suggested that HbA1c could predict the clinical course of patients with DM2 during their hospital stays, and higher level is associated with complications as infectious or cardiovascular diseases.^8,9^ There is evidence that sufficiently supports the relationship of hospital hyperglycemia (capillary blood glucose) with infectious complications, prolongation of inpatient stays, and thrombotic events, not so with HbA1c.^10,11^ There is no clear evidence regarding the clinical events, complications, and outcomes of diabetic hospitalized patients according to the level of HbA1c.^1,5,12,13^ The objective of this study was to describe the clinical and demographic characteristics of diabetic patients hospitalized for nonsurgical diseases, and the relationship with reasons for hospitalization, length of stay, complications (infectious, metabolic, and cardiovascular), readmissions, and treatment modifications at discharge according their HbA1c levels.

## Methods

This was an analytical, cross-sectional, single-center study. After getting the institutional review board approval, medical records were extracted at the Clinica Versalles, a high complexity institution, in Manizales-Colombia, between 2016 and 2017 in the following departments: critical care unit, intermediate care unit, hospitalization service, and the emergency room. Patients who met the following criteria were included: age greater than or equal to 18 years, history of DM2, length of hospital stay greater than 24 hours, at least one assessment by a diabetes specialist (internal medicine or endocrinology) and an HbA1c report during hospitalization.

Pregnant patients and those requiring surgical management were excluded. For the analysis, patients were divided by HbA1c into 4 groups: group 1 = ⩽7%, group 2 = 7.01% to 8.5%, group 3 = 8.51% to ⩽10%, and group 4 = >10%, and for analysis of treatment changes prior to discharge were further divided into 2 groups, those with HbA1c ⩽ 7.5% and those with HbA1c > 7.5%. Statistical analysis was conducted using IBM SPSS Statistics, version 24.0 , licenced for Caldas university. Continuous variables were tested for distribution normality and presented as means ± standard deviation (*SD*), and categorical variables were presented as percentages. The relationship between variables was analyzed with the chi-square test, and a matrix of bivariate correlations was created to analyze the relationship between these variables, as appropriate quantitively.

## Results

In total, 232 patients met the criteria: 136 women (58.6%) and 96 men (41.4%). The average age was 69.7 ± 13.1 years. Of the total number of patients, 146 (62.9%) were aged greater than 65 years. There were no statistically significant differences in HbA1c levels, concerning gender (*P* = .79) or age (*P* = .29). The overall HbA1c average was 7.19% ± 2.03. Of the total, 121 (52.3%) had HbA1c ⩽ 7%, 61 (26.3%) HbA1c between 7.01%–8.5%, 26 (11.2%) HbA1c between 8.51% to ⩽10% and 24 (10.1%) HbA1c > 10%.

### Clinical and demographic characteristics

Among patients, 207 (89.2%) had previous history of cardiovascular disease, 51 (22%) of lung pathologies, 110 (47.4%) metabolic pathologies, and 46 (19.8%) of kidney disease. Through bivariate correlations, an inverse relationship between HbA1C levels and the number of medical records was found (*P* = .002), suggesting that patients with more diseases have more awareness of disease control. Average hospitalization time was 7.5 ± 5.7 days. Of the total, 109 (47%) were hospitalized by 7 days or more, 45 (19.4%) in the emergency department, 204 (87.9%), in the general rooms and 45 (19.4%) in the critical care unit. There was no relationship between groups of HbA1c levels and the length of hospital stays (*P* = .720), nor with the hospital stay in the different hospital departments (*P* = .779) or with complications (*P* = .379). The average body mass index (BMI) was 28.8 ± 5.6, weight was 71.9 ± 15.3 kg, and average height 1.5 ± 0.08 m. No relationship between HbA1c and BMI was found (*P* = 0. 57) nor weight (*P* = .34). A significant difference of socioeconomic status with HbA1c was found, which suggests that the higher the socioeconomic status, worst DM2 control (*P* = .001).

Of the total patients, 11 (4.7%) had no educational level, 73 (31.5%) had 1 to 5 years of education, 62 (26.7%) had 6 to 11 years (Bachellor), and 86 (37.1%) had more than 11 years of education (universitary). It was found that the higher the educational level the lower the HbA1c (*P* = .001). Of the total of patients, 41 (17.7%) were single, 139 (59.9%) were married, and 52 (22.4%) were in free union. Table 1 shows clinical and demographic characteristics.

**Table 1.** Clinical and demographic variable description in accordance with glycosylated hemoglobin.

Glycated hemoglobin	⩽7%	7.01%-8.5%	>8.51%-10%	>10%	Total	*P* value
Sex						
Male	52	22	11	11	96	.79
Female	69	39	15	13	136	
Age						
⩽65 years	41	22	14	9	86	.297
>65 years	80	39	12	15	146	
Education level						
0 years	11	0	0	0	11	.001
1-5 years	33	14	11	15	73	
6-11 years	34	15	10	3	62	
>12 years	43	32	5	6	86	
Civil status						
Single	21	12	4	4	41	.967
Married	74	36	14	15	139	
Free union	26	13	8	5	52	
BMI						
⩽18.5	2	0	0	0	2	.224
18.6-24.9	31	17	9	8	65	
25-29.9	48	20	3	10	81	
30-34.9	22	11	10	4	47	
35-39	10	11	3	2	26	
⩾40	8	2	1	0	11	
Hospitalary services						
Emergency	13	11	10	11	45	.060
Hospitalization	106	52	25	21	204	.554
ICU- INCU	27	14	1	3	45	.116
Two services	25	13	8	7	53	.598
Three services	0	1	1	2	4	.290
Pathological background						
Cardiovascular	107	58	24	18	207	.056
Kidney	27	14	5	0	46	.079
Metabolic	60	33	11	6	110	.092
Respiratory	28	11	7	5	51	.792
Another	32	16	5	7	60	.859
Reasons for hospitalization						
Cardiovascular	67	32	18	15	132	.471
Infectious	28	17	7	8	60	.727
Metabolic	22	2	5	8	37	.004
Another	57	15	13	14	99	.070
Complications						
Cardiovascular	26	15	2	8	51	.161
Infectious	18	7	3	4	32	.880
Metabolic	9	1	1	3	14	.213
Other	17	7	3	3	30	.960
Hypoglycemia						
Present	13	5	4	1	23	.561
Absent	108	56	22	23	209	
Inpatient stay						
<7 days	59	32	17	15	123	.720
⩾7 days	62	29	9	9	109	
Average capillary blood glucose						
<180 mg/dL	80	42	17	12	151	.417
⩾180 mg/dL	32	9	8	5	54	

Abbreviations: BMI, body mass index; ICU, Intensive Care Unit; INCU, Intermediate Care Unit.

### In-hospital characteristics

At the arrival, 132 patients (56.9%) were hospitalized due to cardiovascular disease, 60 (25.9%) by infectious diseases, 37 (15.9%) by metabolic pathologies, and 99 (42.7%) due to other conditions. Those with HbA1c ⩾ 7% were hospitalized for reasons other than acute complications related to their metabolic pathologies (*P* = .004), cardiovascular type the most common. Specifically, 68 (29.3%) patients were admitted for hypertensive disease, and it was found significantly in those with HbA1c levels >8.5% (*P* = .0001) may be with worst control of both diseases. Of total, 27 (11.6%) were hospitalized for congestive heart failure, 16 (6.9%) for myocardial Infarction without ST-segment elevation, 15 (6.5%) for ST-segment elevation myocardial Infarction, and 13 (5.6%) due to unstable angina.

The general average capillary blood glucose measurements during the length of stay were 159.3 ± 51.9 mg/dL. Of this 65.1% had leveled between 70 and 180 mg/dL, 54 (23.3%) had levels above 180 mg/dL, and 3 (9.9%) had at least one hypoglycemia during hospitalization. Table 2 shows hospitalization and glucose level.

**Table 2.** Description of quantitative variables and their relationships with HbA1c in bivariate correlations.

Variable	Minimum	Maximum	Average ± *SD*	HbA1c relationship
HbA1c level (%)	3.7	16.3	7.1 ± 2.03	
Age (years)	29	97	69.7 ± 13.1	*P* = .155
Hospitalization time (days)	1	30	7.52 ± 5.7	*P* = .177
Backgrounds per patient	0	8	2.77 ± 1.4	*P* = .02
Diagnoses per patient	0	8	2.16 ± 1.7	*P* = .388
Average glucometries (mg/dL)	79	466	159.3 ± 51.9	*P* = .827
Hypoglycemia per patient	0	5	0.16 ± 0.5	*P* = .602
Complications per patient	0	5	0.6 ± 0.85	*P* = .32
Number of readmissions	0	9	0.21 ± 0.8	*P* = .953
BMI	16.2	46.9	28.8 ± 5.6	*P* = .576
Weight (kg)	40	120	71.9 ± 15.3	*P* = .344
Size (meters)	1.4	1.8	1.5 ± 0.08	*P* = .506

Abbreviations: BMI, body mass index; *SD*, standard deviation.

### In-hospital complications

Cardiovascular complications were present in 51 (22%) patients, 32 (13.8%) had infectious complications, 14 (6%) metabolic, 30 (12.9%) other complications, and 8 (3.4%) died during hospitalization. Among patients with HbA1c >8.5%, urinary tract infections 24 (10.3%) (*P* = .020) and acute episodes of chronic obstructive pulmonary disease (COPD) 26 (11.2%) (*P* = .003) were significantly more frequent. Globally, no HbA1c relationship was found with inpatient complications (*P* = .151) or mortality (*P* = .367). In the emergency department, 11 (24.4%) patients presented cardiovascular complications, 5 (11.1%) infectious, and 3 (6.7%) metabolic. In the critical care unit service, 13 (28.9%) presented cardiovascular complications, 9 (20%) infectious, and 2 (4.4%) metabolic. There were no significant differences in complications between departments (*P* = .609).

### Discharge treatment and readmission

On admission, 146 (62.9%) had HbA1c levels ⩽7.5% and 86 (37.1%) HbA1C levels >7.5%. No significant differences were found between hospital outcomes in these 2 groups (inpatient stay *P* = .354, readmissions *P* = .686, cardiovascular complications *P* = .975, infectious *P* = .957, and metabolic *P* = .497; Table 3). Of the 145 patients whose illness was considered to be uncontrolled (HbA1c > 7.5%) on admission, 51.2% took oral medication, and 25.6% were discharged with oral medication. The number of patients who brought oral medication plus basal insulin (14%) was reduced to 7% at the time of discharge. Patients who only had basal insulin on admission were 10.5%, and for discharge 19.8%. Patients with basal insulin plus prandial insulin on admission totaled 16.3%, and at on discharge totaled 37.2%. Table 4 shows treatment changes, in accordance with HbA1c levels.

**Table 3.** Hospital variables by HbA1c.

Glycated hemoglobin	<7.5%	⩾7.5%	Total	*P* value
Inpatient stay				
<7 days	74	49	123	.354
⩾7 days	72	37	109	
Readmissions				
None	125	77	202	.686
1-2 times	19	8	27	
⩾3 times	2	1	3	
Inpatient complications				
Cardiovascular	32	19	51	.975
Infectious	20	12	32	.975
Metabolic	10	4	14	.497
Other	21	9	30	.290

**Table 4.** Treatment table.

HbA1c level	HbA1c < 7.5%				HbA1c ⩾ 7.5%			
Scheme	Admission	%	Discharge	%	Admission	%	Discharge	%
Oral	55	37.7	36	24.7	44	51.2	22	25.6
Oral + basal insulin	28	19.2	10	6.8	12	14	6	7
Basal insulin	22	15.1	14	9.6	9	10.5	17	19.8
Basal + rapid insulin	30	20.5	65	44.5	14	16.3	32	37.2
No medication	11	7.5	−	−	7	8.1	−	−

After patient discharge and in the following 3 months, 27 (11.6%) had 1 or 2 readmissions, 3 (1.3%) had 3 or more readmissions. No relationship was found between the number of readmissions and HbA1c levels (*P* = .609).

## Discussion

For the majority of the world’s population, hospitalization is an important period of time, as it impacts both morbidity and mortality. In this study, HbA1c levels seem not to influence hospital length and some outcomes. Currently, there is strong evidence regarding the impact of acute hyperglycemia on these hospital outcomes, but there is no clear information on the effect of HbA1c levels on them.^10^ The results of this investigation support the concept that HbA1c levels do not modify outcomes. However, it is clear that elevated HbA1c levels are accompanied by hospitalizations, owing to multiple comorbidities and to infectious diseases.^14^

One study performed in Colombia by Osuna et al^5^ described a population similar to this, in which 65.6% of patients were aged more than 65 years and 66.8% were women. In-hospital length was not shown to be dependent on HbA1c levels in those with HbA1c levels above 9%. The proportion of patients who developed hypoglycemia in both studies was similar, with 9.9% and 11%.

In Australia, Lee et al^12^ found that the most frequent cause of hospitalization was infectious disease in 42% of cases, mainly due to respiratory and soft tissue infections, followed by 14% caused by cardiovascular disease. This situation is the opposite of that described in this study, as the most common cause of hospitalization was cardiovascular disease, but more infectious diseases in patients with poor control. Regarding complications which arise during hospitalization, Bonamichi et al^1^ from Brazil describe that those with HbA1c levels between 7.3% and 12.4% had higher numbers of infectious complications, with lung infection as the most probable cause, with 50%, and septic shock as the second, with 15%. A study conducted by Méndez-García et al^15^ found that with 428 diabetic Mexican patients hospitalized, there was an increase in mortality for those with HbA1c levels ⩾8%, the average level in patients who died was 9.1%, and their average glucose level was 158 mg/dL, which was higher in those who survived. Gonzales-Grández et al^16^ described in a study conducted in Peru, with 424 patients, of whom 32.3% were admitted without any treatment at all, 41.3% were admitted with oral medication, 10.9% entered with oral medication plus basal insulin, and 4.9% entered with only basal insulin. These findings are quite different from those of this investigation. However, despite having similar ages, patient lifestyles, social conditions, and health systems are nonsimilar, which could explain this dissimilarity. A fundamental finding of this study was the change in treatment between admission and discharge, as there was an increase in patients with insulin therapy at discharge, even in patients whose baseline HbA1c was controlled. This result reflects the continuation of patient treatment, with which it was possible to obtain adequate inpatient control of their illnesses.

No relationship between HbA1c levels and readmission numbers was identified. Montero Pérez-Barquero et al,^17^ however, did discover said relationship in their study, after discharge, when patients were monitored for 12 months, and 56.4% of patients were readmitted and/or died. In addition, it was found that 43.6% were not readmitted or died during that period. The aforementioned study had a much longer monitoring period, 12 months, compared to 3 months in this study.

Limitations of our study include a single-center study, the number of patients with high levels of glycosylated hemoglobin was low, the duration of the patients’ illness was unknown, and it was not possible to establish a timeline for capillary blood glucose control during hospitalization and chronic control appointments.

It is necessary to further analyze evidence that suggests the extent to which glycosylated hemoglobin influences the inpatient outcomes of patients with diabetes, so as to determine the scope of the interventions or deferred interventions necessary.

## Conclusions

In diabetic adult patients with nonsurgical diseases, the HbA1c seems to have no impact on the length of hospitalization, nor on the number of readmissions or complications. Strikingly, 65% of the patients had in-hospital glucose level in goals, and there were few cases of hypoglycemia, is not clear if high HbA1c levels with normal in-hospital glucose could change this aspect or be indifferent. Regarding the treatment, it is worth highlighting the change in the management of diabetes, generating new treatment schemes for the outpatient scenario. In our cohort, the level of HbA1c above 7% were admitted with significantly increased cardiovascular morbidities and higher than 8.5% to infectious diseases.

### Limitations

1. Information regarding DM2 diagnosis evolution time was not found.
2. Only capillary blood glucose reported in virtual clinical histories were included in the study, thus the real number of glucometries performed during hospitalization is not accurately reflected.
